# Clinical challenges of classification based targeted therapies for non-specific low back pain: What do physiotherapy practitioners and managers think?

**DOI:** 10.1016/j.math.2014.11.008

**Published:** 2015-06

**Authors:** Liba Sheeran, Philippa Coales, Valerie Sparkes

**Affiliations:** Cardiff University, School of Healthcare Sciences, Ty Dewi Sant, Heath Park, Cardiff, CF14 4XN, UK

**Keywords:** Classification, Non-specific low back pain, Subgroups, Health practitioner opinions

## Abstract

**Background:**

Classification of non-specific low back pain (NSLBP) was recommended to better target care and so maximise treatment potential. This study investigated physiotherapy practitioners' (PPs) and managers' (PMs) views, experiences and perceptions of barriers and enablers for using classification systems (CSs) to better target treatment for NSLBP in the NHS primary care setting.

**Design:**

Qualitative focus group and interviews.

**Methods:**

Data from semi-structured interviews of three PMs and a focus group with five PPs, considered local opinion leaders in physiotherapy, was thematically analysed.

**Results:**

Five themes emerged (i) CS knowledge: PPs and PMs were aware of CSs and agreed with its usefulness. PPs were mostly aware of CSs informing specific treatments whilst PMs were aware of prognosis based CSs. (ii) Using CSs: PPs classify by experience and clinical reasoning skills, shifting between multiple CSs. PMs were confident that evidence-based practice takes place but believed CSs may not be always used. (iii) Advantages/disadvantages of CSs: Effective targeting of treatments to patients was perceived as advantageous; but the amount of training required was perceived as disadvantageous. (iv) Barriers: Patients' expectations, clinicians' perceptions, insufficiently complex CSs, lack of training resources. (v) Enablers: Development of sufficiently complex CSs, placed within the clinical reasoning process, mentoring, positive engagement with stakeholders and patients.

**Conclusions:**

PPs and PMs were aware of CSs and agreed with its usefulness. The current classification process was perceived to be largely influenced by individual practitioner knowledge and clinical reasoning skills rather than being based on one CS alone. Barriers and enablers were identified for future research.

## 1 Background

Low back pain (LBP) is a highly prevalent and disabling pain disorder impacting on people's health and quality of life world-wide (Ehrlich, 2003). Each year 6–9% of adults consult their GPs about back pain (Dunn and Croft, 2006; Jordan et al., 2010) which in the majority of cases is non-specific (NSLBP) (Waddell, 2004). Most available treatments have low to moderate short lasting benefits (Pransky et al., 2010; Patel et al., 2013), suggested to result from the NSLBP heterogeneity and variable treatment response (O'Sullivan, 2006). Identification of subgroups to better target care and maximize treatment potential is a pressing research priority (Costa et al., 2013) and was a key research recommendation in a recent National Institute of Clinical Excellence (NICE) Guideline for Early Management of Persistent Non-Specific Low Back Pain (Savigny et al., 2009). Whilst there is some evidence that health practitioners recognize the multifactorial nature of back pain and its unlikeliness to respond to a single management approach, the consensus on how to subclassify NSLBP is lacking (Kent and Keating, 2004, 2005). This may pose a potential challenge for implementation of classification system (CS) based treatments for NSLBP in clinical practice. Gathering views from and knowledge of local health service groups and clinical opinion leaders is thought critical for successful implementation of guidelines and research into clinical practice (van Tulder et al., 2002a; Cote et al., 2009). To the best of our knowledge, opinions of health service groups such as the UK National Health Service (NHS) physiotherapists, whose workload predominantly consists of managing patients with NSLBP, has not previously been investigated.

The aim of this study was to evaluate a group of UK NHS physiotherapists and managers experiences and perceptions of barriers and enablers for implementation of classification systems to better target treatment for NSLBP in the NHS primary care setting.

## 2 Method

### 2.1 Design

A qualitative phenomenological approach, using semi-structured interviews and a focus group, was used to gain deeper understanding and knowledge through exploring personal accounts and people's experiences (Petty et al., 2012a, 2012b) (Stenfors-Hayes et al., 2013). Ethical approval was gained from the Research Ethics Committee for Wales (Reference number 10/MRE09/28).

### 2.2 Participants

Critical case sampling of Primary Care Physiotherapy Practitioners (PP) and Managers (PM) considered by their peers as the opinion leaders within the two Health Boards in Wales, UK were recruited. Opinion leaders (also termed as the Informal Opinion Leaders) are considered to be credible and representative of other team members' attitudes and behaviours and therefore considered to hold views more representative of wider clinical opinion (Flodgren et al., 2011). The selection process involved the informant method (Valente and Pumpuang, 2007) where the physiotherapy staff from both Health Boards were asked to nominate individuals they consider as opinion leaders. In addition, the respective Heads of Therapies responsible for staffing, organisation and delivery of back pain management care pathways were consulted to contribute to the nominations. All nominated individuals were sent an invitation letter and those expressing interest in participating were sent an information sheet and were contacted by the researcher (LS) to further clarify any questions regarding their participation. Written informed consent was gained prior to data collection.

### 2.3 Data collection

Data from the PPs was obtained via a focus group. The PMs undertook individual semi-structured interviews as it was not logistically possible to select a single date suitable to all managers. In addition, in line with guidance on conducting focus groups (Stenfors-Hayes et al., 2013), it was felt that PMs responsible for service delivery in the neighbouring Health Boards would speak more openly about their views and experiences in an interview setting rather than in a focus group environment. Due to clinical demands a second focus group only recruited one PP who was subsequently interviewed individually. The focus group took place on Cardiff University premises and the interviews were conducted in PMs' own offices for convenience reasons. The researcher conducting the focus group and interviews (PC) was known to all participants as impartial to opinions on classification of back pain ensuring credibility. A topic guide with questions (Table 1) was constructed from a review of current literature on LBP classification exploring clinicians' understanding of LBP classification and targeted therapies (Kent and Keating, 2004, 2005). The question development included a pilot where two clinicians and two university academics were asked to comment on clarity and flow of the questions Final structure and the flow of the questions was once more piloted using the same individuals. Finally, in line with an iterative approach, which entails cycles of simultaneous data collection and analysis (Lingard et al., 2008), questions used within the PPs focus group were further modified for the managers' interviews. Prior to the focus group/interviews, their purpose was explained and definitions given for “NSLBP”, “classification” and “classification-guided targeted therapies”. The focus groups and interviews were audio-recorded and transcribed verbatim by a third party. A research observer took field notes during all interviews and the focus groups. Blinded transcription identified the individuals within the focus group to enable the analysers to relate comments to participants. Each participant was allocated a unique code to anonymise participants to the researchers analysing the transcripts. Member checks were carried out where all participants were sent the relevant focus group or interview transcripts to check their authenticity and completeness (Pope et al., 2000). A single focus group participant raised an issue related to wording. This was rectified within the transcript.

### 2.4 Data analysis

**Fig. 1:**
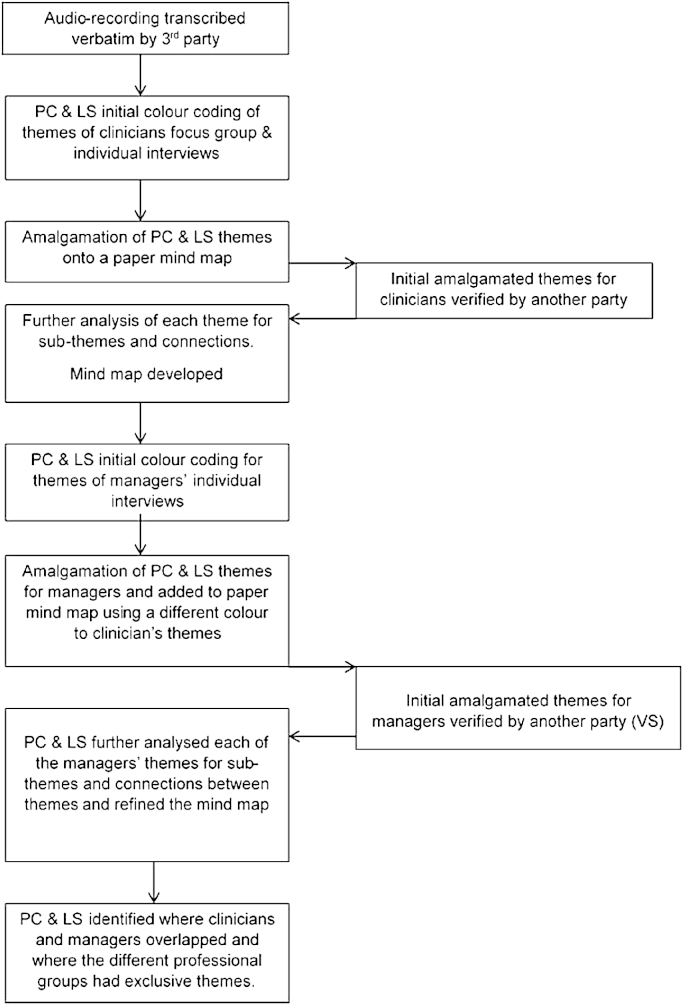
Flow chart of thematic data analysis.

The intention of this study was to provide rich thematic description (Braun and Clarke, 2006) of predominant opinions that physiotherapy practitioners and managers have about classifying NSLBP. The data were independently analysed by the researchers (PC, LS, VS) through inductive thematic analysis, using an editing approach (Riley and Hawe, 2005). Thematic analysis is detailed in Fig. 1. Following an independent reading and re-reading of the transcripts by the researchers, initial analysis of the focus group and interviews involved colour coding into meaningful groups within the printed transcriptions by LS and PC independently. The codes were guided by (i) the links to the questions and prompts used for further discussion and (ii) prevalence of repeated phrases and words across the data set (Braun and Clarke, 2006). All extracted data was independently coded, collated and grouped within separate mind maps to form the basis for generating the initial themes. The codes were verified by a third researcher (VS), who also acted to resolve any analytical disparities between the two main researchers. The two researchers' initial themes were then amalgamated by creating a detailed mind map, which was jointly analysed by PC & LS for the main themes, sub-themes, potential new connections and all extract of data coded within each theme. The final overarching themes were again verified by VS. The final part of the analysis identified where PPs and PMs overlapped and where the different groups had exclusive themes. During the analysis the researchers were open to possible unexpected themes resulting from the questions.

## 3 Results

Out of 10 potential participants approached, one was unable to attend any of the available focus group or interview dates due to family emergency; therefore 9 participants took part in the study. Key participant characteristics are detailed in Table 2. They were mostly male with average 24.1 (+/− 9.2) years of clinical experience.

Common themes that emerged from the focus group and interviews are summarized in Table 3. The coverage, referring to the number of times PPs and PMs commented within each of the identified themes and the proportionate distribution (volume) of the themes discussed within the focus group and the interviews is shown in Table 3. All PPs and PMs made comments within all of the identified themes. Knowledge and current use of LBP classification themes received similar attention by PPs and PMs (Table 3). Both PPs and PMs made proportionately more comments on the perceived advantages rather than the disadvantages of CS based targeted therapies. PPs discussions mostly centred around the barriers for adopting classification (29% of the total focus group), whilst PMs mostly focussed on enablers for implementation (34%) (Table 3).

### 3.1 Knowledge of CSs for LBP

Both PPs and PMs viewed LBP as a complex multi-dimensional disorder with classification providing a route to guide management. *“Back pain is complex and different classifications allow for targeting treatment more effectively*” [P07] *“back pain has different triggers … classification helps to guide interventions appropriate to subgroups”* [P02], All *“Classification aims to provide the right treatment for the right patient”* [P07]. PPs and PMs were aware of different ways to classify back pain acknowledging its complexity “subgrouping back pain becomes incredibly complex, because patients may have a clinical pattern but they may also have a psychosocial pattern and trying to suit a classification to a patient is actually very difficult [P01]). PPs were mostly aware of the mechanism-based CS “classification helps to establish what is it that needs to change to prevent someone carrying on in that circle [P04] “*I know McKenzie and O'Sullivan's, movement impairment …*” [P01, 02, 03]. PMs were mostly aware of patho-anatomy based CS in the form of triage and those concerned with referral pathways and prognostic indicators “*STarTBack … sort of a risk stratification*” [P07]; *“the majority are psychosocial non-medical systems”* [P08].

### 3.2 Current use of CSs to target therapies for LBP

Both PPs and PMs reported using a triage system to exclude serious pathology *“my reasoning starts with serious pathologies, then nerve root symptoms and then … non-specific”* [P04, P05,P07]. For non-specific LBP, PPs report to use *McKenzie* [P01, P04 FG; P06], *“O'Sullivan's, Movement Dysfunction and Maitland* [P01, P04], “psycho-social yellow flags” [P07]. PPs agreed with a statement made by P05 *“classification system that covers the biomedical, psychological and social domain* … *is the holy grail”* but believe that most CSs are uni-dimensional *“… no one classification fits all*…*”* [P05]. PPs reported to “*shift between different classifications … depending on what is in front of us, because of the complexity of that group*…*”* [P03, P01, P02]. The process of classification appeared to be dependent on individual clinician's experience, and clinical reasoning process employed *“*…*clinical reasoning skills are really important* … *and it depends on how you're trained and* … *fed through the system*…*”*[P01]. Interestingly, PPs saw classification as an unconscious process, *“I don't use classification consciously, it's more of a subconscious process …*” [P04,] Varied abilities in clinical reasoning skills appear to figure; currently combated by managing the allocation of the caseload based on complexity of patients and clinical experience of individual practitioners …”*more junior physiotherapist allocated to “simple” back pain cases … more experienced clinicians see more complex cases”* [P04]. There was uncertainty about the actual process of classification currently used *“I'm trying to think how therapists might classify, I'm not certain actually”* [P04]. Resources available and patients' initial response to treatment also appears to also play part in the process of classification *“… subclassify almost backwardly … because we quite often put them into the group setting when we feel that they haven't actually done that well with other therapies” [*P03*]*.

PMs believed that physiotherapists are *“much better briefed about biopsychosocial classification than other professional groups”* [P07] and are confident in their clinicians providing evidence-based practice. PMs also believe however that besides triage, classification based management is not common practice *“I don't think classification is used commonly across the department and the service*” [P07]. PMs were uncertain on how patients were allocated to treatment options *“*…from my experience there is a problem with how patients find the route through the pathway… what is tending to happen is they (patients) go through the system in a linear progression ending up in secondary care or in pain teams as failed back pain” [P07].

### 3.3 Perceived advantages and disadvantages of CSs to target therapies for LBP

Both PPs and PMs perceived classification in a positive light: *“targeting the right patients for the right therapy early”* [P03, P07]. An important advantage perceived by PPs was bringing treatments to patients that are specific and individual to their problem, which would *“more likely improve outcomes”* [P07, P02, P03] and *“win patients' trust”* [P07]. Formalized classification was perceived as *“giving a language”* for teaching and development of clinical reasoning skills [P04]. PMs perceived classification potentially facilitating *“a cost effective, resource efficient service”* [P07, P08]. Among the disadvantages, some saw that CSs were *“developed for research purposes”* [P05], *“clinical usefulness is questionable”* [P01] and *“not evidence based”* [P02, P03]. Back pain was also viewed as being too complex to classify *“*…*you can't actually put people into a set box and only treat them by that approach*…” [P07] … *“not everybody fits in a box … it's recognising that you might have to have the box open so you can move them (patient) in and out of it”* [P01]. PPs also view classification as *“..guru led”* [P03, P05], ”*potentially compromising clinical reasoning skills*” [P01,05]. According to P03 “*Junior physios almost become protocol-led rather than generating their own clinical reasoning* … *they (junior clinicians) use treatment protocols quite systematically and when taken away they have no structure whatsoever*” [P03]. Interestingly, CS based targeted therapies were seen by some as a move away from patient centred care “*danger of … CS is in making (the) patient fit the system”* [P05]. Lastly, adopting a single classification approach was viewed as a threat to therapeutic diversity *“lose the dimensions, the depth of what your department can offer”* [P01].

### 3.4 Barriers for adopting classification to target therapies for LBP

The main barrier was the perceived inadequacy of the current CSs seen *as “uni-dimensional, lacking complexity”* [P01, P05] and based on judgement of a few people considered as experts: “some CSs are … clinical judgement led.. with somebody saying this is my treatment approach and then the classification system fits around that … author bias is massive … a danger that we get … guru lead practice by the back door really …” [P05]. Rotational staff were perceived by some PPs as a barrier “rotational staff coming with their own ideas, any new approach would get quite quickly diluted unless it was very well regulated” [P03]. Other barriers included varied levels of staff experience, lack of time and resources to up-skill junior clinicians [P01, P05]. Others perceived junior clinicians as open to new ideas *“for more junior members with less baggage it would be easier to accept new treatment methods”* [P07].

PMs hoped *“lack of time would not be an obstacle”* [P08] but viewed *“resource and organizational issues related to shortages of static staff and retention … a potential barrier”* [P07, P08]. Both PPs and PMs perceived other health professions as a potential obstacle to integrating CS into clinical practice “others (health professionals) may be reluctant to move away from the traditional medical model and take on board what the evidence shows” [P06]. Patients' expectations fuelled by mixed messages from different professionals were also seen as potential barriers, *“..some (health) professionals say patients needs surgery and some say not … this will have a significant impact on patients' psychology, …”* [P06]. The main obstacle for the managers was the process of integrating CS into the currently existing complex referral systems [P07] and concerns about *“how patients find their way through the multiple pathways with individual professions protecting their own turf and inter-professional agendas”* [P07, P08].

### 3.5 Enablers for adopting classification to target therapies for LBP

To ensure successful adoption of CSs for targeted care of LBP, PPSs expressed that CS “*needs to be placed within the clinical reasoning process and has to have some element of flexibility”* [P01, P02], *“staff need to be aware of the importance of sub-classification and engage with the process”*[P06]. *“The system must be multi-faceted”* [P02] *… of sufficient complexity and incorporating all three bio-psycho-social domains”* [P01, P05]. Creating a supportive environment for training and development was perceived as essential “a good training package … to provide a practical solution to dealing with patients clinically needs to be in place [P06]. All participants stressed that positive engagement through dialogue with all health professions including rheumatologists, GPs, spinal surgeons and commissioners was essential. P08 and P05 stated *“if we got the referral system right and the classification right, then the GP should be saying the physio has got to be your first port of call”*. Process evaluation was seen as a potential way to engage staff and stakeholders “..it needs evaluation so that … we learn as therapists as an organisation about the effectiveness of those different (treatment) arms” [P04]. All participants agreed that the key enabler for successful integration of CSs was engaging with patients through education, and managing their expectations.

## 4 Discussion

Classification to better target care for LBP has been a top research priority for over 15 years (Pransky and Cifuentes, 2009; Costa et al., 2013). But how acceptable classification-based targeted therapies for LBP are to the physiotherapy service in the UK is currently unclear. This study presents for the first time the UK physiotherapy practitioners and managers frank views and perceptions of LBP classification to targeted physiotherapy treatments. The findings serve as an important learning point for research to develop CS based targeted interventions that are feasible and acceptable to the health practitioners delivering them.

### 4.1 Knowledge of CSs for LBP

The UK PPs and PMs in this study largely viewed classification of LBP in a positive light and saw its importance given the complexity and heterogeneity of the caseload. This is in line with views of the Australian health practitioners who perceived LBP as multi-factorial requiring a different type of management (Kent and Keating, 2004). PPs in the current study were most aware of the multi-dimensional classification system (MDCS) (O'Sullivan, 2005) and mechanical diagnosis approach (McKenzie, 1989), whereas PMs were most aware of the StarTBack Tool (Hill et al., 2008). This awareness may be a result of the adopted dissemination practices through established training postgraduate programmes (www.mckenzieinstitute.co.uk) and innovative web-based platforms (e.g. www.pain-ed.com, www.keele.ac.uk/sbst)known to influence permeation of research into clinical practice (van Tulder et al., 2002b). As well as the quality and relevance of the evidence base, clinical awareness of a particular CS may also be driven by the level of its relevance to the particular job role. In this study PPs were mostly aware of CSs specifically guiding the actual therapies (McKenzie, 1989; O'Sullivan, 2005) whilst PMs were mostly aware of CSs concerned with stratifying LBP into relevant care pathways, such as STarTBack (Hill et al., 2008).

### 4.2 Current use of CS to target LBP therapies

This study has shown that PPs tend to use a range of different CSs with limited consensus on how to best classify NSLBP. This is in agreement with Kent and Keating's (2005) postal survey that found little consensus on the specific clinical signs of NSLBP subgroups across professional disciplines in Australia. Through exploring views and attitudes of the opinion leaders considered capable of driving and facilitating change (Flodgren et al., 2011), this present study further advanced this knowledge by identifying potential reasons for the lack of consensus. One such reason expressed by the PMs was linked to the geographical challenges with practitioners working in very diverse city and rural areas. Working in large geographical areas was in fact shown to be a barrier for developing shared community practice and delivery of a unified service (Stevenson et al., 2004). The complexity of LBP may be another reason for the lack of consensus. This study revealed that physiotherapy practitioners resort to a range of different strategies to deal with the complexities of LBP including allocating complex cases to experienced staff, swapping between different CSs in response to the changing clinical patient profile, and “backwardly sub-classifying’, where treatments are determined by failure to respond to previous treatments. This is an interesting observation that reflects challenges faced by the NHS physiotherapists in their attempts to adopt research based practice. Previous research noted that clinicians expressed concerns about their level of expertise and training when it comes to targeted care (Corbett et al., 2009). Given that stratified models of care are being explored in a range of other conditions (Hewner et al., 2014), a review of the current healthcare undergraduate and postgraduate curriculum may be warranted to reflect this drive towards new delivery of healthcare.

### 4.3 Advantages, disadvantages, barriers and enablers for adopting CS based targeted therapies for LBP

Overall in this study, CS to target care better for LBP was viewed as advantageous facilitating patient centred care, ensuring efficacy and better use of resources. However, there was some reluctance to its adoption, highlighted by the large proportion of discussions centred around the barriers and disadvantages (Table 3). Classification was perceived by some as a potential threat to professional autonomy and clinical reasoning skills refinement; a research exercise of limited clinical relevance with need for substantial training and development of complex referral pathways. Resistance from other professional groups has also been noted as a potential obstacle. Interestingly, these findings mirror barriers associated with adopting evidence based physiotherapy practice (Jette et al., 2003; Stevenson et al., 2004) back pain research into primary care (van Tulder et al., 2002b) and doctors following clinical guidelines (Mehta, 2004). Crucially, this study also identified PPs' and PMs' perceived enablers for CS targeted therapies for LBP, including the need for CS incorporation within the current clinical reasoning processes, adequate training resources, dialogue between all health professional groups, commissioners and patients. Consideration of adopting more than one CS to satisfy LBP complexity and the NHS requirements has also been perceived as enabler, which is in line with research exploring translation of stratified models of care for LBP (Foster et al., 2013).

### 4.4 Study limitations

Due to the small scale and limited funding for this study, one limitation was the relatively small sample sufficient for a single focus group and three interviews. Although this is a limiting factor in terms of the representatives of this study findings, utilizing critical case sampling strategy to recruit individuals nominated by their peers as the key opinion leaders, considered to hold views representative of other team members (Flodgren et al., 2011), led to gaining views representative of the wider clinical opinion (Valente and Pumpuang, 2007). Neverthelss, we acknowledge that the results of this study need to be considered in light of its relatively small size with further research to carry out in other UK NHS primary care settings for the findings to be fully representative.

### 4.5 Future research

Future research needs to also include gaining views on classifying NSLBP across disciplines and from other health professionals involved in the management of patients with NSLBP as well as the patients themselves. In line with complex intervention development guidelines, the results of this study are currently being used to refine a classification based targeted functional cognitive therapy (CB-CFT) specifically developed for people with non-specific LBP treated in the NHS primary care setting.

## 5 Conclusion

The study findings indicate that physiotherapy practitioners and managers have clear knowledge of different classification systems and generally agree with its usefulness to guide LBP management. Whilst the practitioners see CSs usefulness to inform the specifics of the treatment delivered, managers have a much broader view on the service delivery as a whole. Both the physiotherapy practitioners and managers believed that a targeted CS based approach is utilized for the management of LBP, although how patients are classified is driven largely by the individual practitioners' knowledge and level of clinical reasoning skills rather than having a standardized CS approach in place. A range of barriers and enablers were identified for future research.

## Figures and Tables

**Table 1:** Topic guide for (a) Physiotherapy Practitioners (b) Physiotherapy Managers.

**(a) Topic Guide for Physiotherapy Practitioners**
What do you see classification for LBP to be? What is your understanding of LBP classification/CS based targeted physiotherapy treatments?
What do you see as your role in delivering targeted treatments? Do you use classification to target physiotherapy treatments? What is your role in delivery of CS based targeted treatments? Do you refer to relevant CS based targeted treatments? What is the role of the Physiotherapy Department as a whole implementation/delivering targeted treatments to manage LBP?
What are the barriers/challenges? What do you know about CS based targeted treatments for LBP? How can patients' access CS based targeted treatments? How do you feel about delivering CS based targeted treatments? (your autonomy, expertise, knowledge) What are the managers' attitudes and perceptions? (costs, organization, fit with other services) What are patients' attitudes and perceptions?
What issues are important when delivering/implementing targeted treatments to manage CLBP? What are you confident/not confident about managing?
What are the difficulties/barriers of delivering/implementing CS based targeted interventions?
What might help you to deliver/implement CS based targeted interventions more effectively?
**(b) Topic Guide for Physiotherapy Managers**
What do you see classification of LBP to be? What is your understanding of LBP classification/CS based targeted physiotherapy treatments?
What do you see as your role in implementing targeted treatments? Do your departments use classification to deliver targeted physiotherapy treatments? What is your role in delivery/implementation of targeted treatments? Do your practitioners refer to relevant CS based targeted treatments? What is the role of the Physiotherapy Department as a whole in implementation/delivery of targeted treatments to manage LBP?
What are the barriers/challenges? What do you know about CS based targeted treatments for LBP? Are patients aware of targeted treatments and how do patients access targeted treatments? How do you feel about implementing classification to target physiotherapy treatments? (costs, fitting in with other services, confidence in clinicians to be able to deliver it?) What are the clinicians' attitudes and perceptions? (clinicians' autonomy, expertise, knowledge) What are patients' attitudes and perceptions?
What issues are important when delivering/implementing CS based targeted treatments for LBP? What are you confident/not confident about managing?
What are the difficulties/barriers of delivering/implementing CS based targeted interventions?
What might help you to deliver/implement CS based targeted interventions more effectively?

Key: CS=Classification System.

**Table 2:** Participant (P) characteristics.

Participant code	Gender male (M) Female (F)	Years clinical experience	Qualifications	Physiotherapy practitioner (PP) Physiotherapy Manager (PM)	Interview (I) Focus group (FG)
P01	F	39	PhD, MSc	PP	FG
P02	M	19	MSc	PP	FG
P03	M	12	MSc	PP	FG
P04	M	22	BSc (Hons)	PP	FG
P05	F	25	BSc (Hons)	PP	I
P06	M	13	MSc	PP	FG
P07	M	32	Grad Phys Dip	PM	I
P08	M	33	Grad Phys Dip	PM	I
P09	M	20	MSc	PP	FG

**Table 3:** Prevalence of counts raised by Physiotherapy Practitioners and Managers within each theme.

Themes	Physiotherapy practitioners (*n* = 6)		Physiotherapy managers (*n* = 2)	
	Coverage: number of participants commented	Volume: number of coded single line text units (%)	Coverage: number of participants commented	Volume: number of coded single line text units (%)
Knowledge of CSs	6	144 (13%)	2	102 (13%)
Use of CSs to manage NSLBP	6	204 (19%)	2	150 (19%)
Advantages of CSs to manage NSLBP	6	78 (7%)	2	59 (8%)
Disadvantages of CSs to manage NSLBP	6	30 (2%)	2	7 (0.9%)
Barriers for adopting CSs	6	309 (29%)	2	180 (23%)
Enablers for adopting CSs	6	297 (26%)	2	264 (34%)

Key: CS = classification system, NSLBP = non-specific low back pain.
